# A qualitative study on the development and rectification of advanced medical students’ misconceptions about the physiological electrocardiogram (ECG)

**DOI:** 10.3205/zma001280

**Published:** 2019-11-15

**Authors:** Mathias Trauschke

**Affiliations:** 1 Leibniz University of Hannover, Institute for Science Education Research (Biology Education), Hannover, Germany

**Keywords:** ECG (misconceptions), Model of Educational Reconstruction (MER), Conceptual Metaphor Theory

## Abstract

**Purpose:** The study takes a qualitative and explorative approach to capture the concepts that German medical students have of the physiological electrocardiogram (ECG) which they acquired during their preclinical training. These concepts are then considered for possible misconceptions. Afterwards a theory-based intervention which allows subjects to see the connection of the curve progression with the spatial spreading of excitation (animated vector loop) is put to the test.

**Methods:** In the course of a diagnosis of learning potentials, individual students participated in problem-centred, guided interviews. The developed intervention was tested in separately conducted teaching experiments using thinking aloud protocols. The data evaluation was done through qualitative content analysis. Based on the conceptual metaphor theory, conceptions and their underlying embodied cognition were analysed.

**Results: **One of the subjects’ typical misinterpretations is taking the progression of the ECG tracing for a mere increase and decrease of the myocardium’s electrical activity, rather than connecting it with its spatial and temporal aspects. The data evaluation has shown that the newly developed theory-based intervention can lead to re-learning. Reconstructed metaphorical concepts illustrate this process of understanding. It is exemplarily shown how, through the course of the interviews, students are enabled to appropriately explain ECGs as the two-dimensional representation of the spatial excitation propagation in the heart.

**Conclusion: **By capturing typical misconceptions of the physiological electrocardiogram and demonstrating interventions that support learning, the study provides a contribution to comprehensive learning which can be used in the basic education of medical students.

## Zusammenfassung

**Zielsetzung:** In dieser qualitativ-explorativen Studie werden Vorstellungen von Studierenden im klinischen Studienabschnitt der Humanmedizin über das im präklinischen Studienabschnitt behandelte physiologische Elektrokardiogramm (EKG) erfasst und auf mögliche Fehlvorstellungen untersucht. Ferner wird eine theoriegeleitet entwickelte Intervention zur Verknüpfung von Kurvenverlauf und der dadurch repräsentierten räumlichen Ausbreitung von Erregungsmustern (animierte Vektorschleife) erprobt.

**Methodik: **Im Zuge einer Lernpotenzialdiagnostik wurden Studierende einzeln mithilfe problemzentrierter, Leitfaden-gestützten Interviews befragt. Die entwickelte Intervention wurde mithilfe einzeln durchgeführter Vermittlungsexperimente unter Nutzung von lautem Denken erprobt. Die Datenauswertung erfolgte durch qualitative Inhaltsanalyse. Vorstellungen und die diesen zugrundeliegenden basalen Kognitionen wurden im Lichte der Conceptual Metaphor Theory analysiert.

**Ergebnisse:** Eine charakteristische Fehlinterpretation besteht darin, dass Probanden die räumlichen und zeitlichen Aspekte der Erregungsausbreitung nicht mit dem Verlauf des EKG verknüpfen und im Kurvenverlauf lediglich eine Zu- und Abnahme von elektrischer Aktivität im Myokard erkennen. Wie die Auswertung der Vermittlungsexperimente zeigt, kann eine theoriegeleitet entwickelte Intervention zum einem Umlernen führen. Anhand von rekonstruierten Metaphernkonzepten wird dieser Verstehensprozess exemplarisch illustriert und aufgezeigt, wie Studierende im Verlauf der Interviews ein EKG als zweidimensionale Repräsentation der räumlichen Erregungsausbreitung im Myokard angemessen erläutern können.

**Schlussfolgerung:** Durch das Erfassen typischer Fehlvorstellungen zum physiologischen Elektrokardiogramm und das Aufzeigen lernförderlicher Interventionen liefert die Studie einen Beitrag zum verstehenden Lernen, der in der physiologischen Grundausbildung von Medizinstudierenden genutzt werden kann.

## 1. Introduction

The differences in potentials shown by the ECG result from the excitation of the heart. The ECG can give information on the position of the heart as well as on frequency, rhythm and spatial excitation propagation. The direction and the magnitude of the potentials vary throughout the excitation propagation which can be visualized by vectors. The vector sum, calculated from the single vectors, produces a typical loop-shaped graph in the course of the excitation. The chronological sequence of the vector – projected on the frontal, sagittal or horizontal plane – can be visualized in the ECG [1]. The ability to read and interpret an ECG is one of the key competences that students of human and veterinary medicine must acquire. However, acquiring this competence poses a particular challenge [2]. A number of studies on the teaching and learning of competences and abilities needed for ECG interpretation are therefore subject of current research in education research [2], [3], [4], [5], [6]. All of the research approaches refer mainly to the competence in interpreting pathological ECGs in the clinical part of the medical training. Students’ basal concepts of the basics of the physiological ECG are less considered. Thus, conducting a qualitative study, students’ understanding of ECGs was recorded in order to identify possible learning difficulties and to explore theory-based developed learning opportunities. To do so, the concepts students of human medicine have of physiological ECGs were captured by using guided interviews [7] and qualitative content analysis [8]. A specially designed intervention (video animation, see attachment 1 ) was tested in teaching experiments [9].

Abstract information – like the varying directions of potentials represented in the ECG [1] – is cognitively extrapolated by employing conceptual metaphors [10]. The conceptual metaphor theory [11] used in the research of concepts in science education research was therefore used as an instrument of analysis [12] to model thinking processes. The reconstructed concepts drawn from the interviews and the teaching experiments were consequently interpreted by using systematic metaphor analysis [13]. The paper describes typical misconceptions of the physiological ECG and identifies a possible way of learning appropriate concepts of the abstractly visualized information on the directions of excitation in an ECG.

## 2. Theoretical framework

This research approach is based on the holistic approach known as cognitive linguistics, which states that syntax and semantics are intricately bound to each other. Linguistic phenomena are therefore of analytical interest in order to model conceptual structures [14]. 

The conceptual metaphor theory is used [10], [11], [12] to analyze the understanding (and non-understanding) of a physiological ECG and to make predictions about interventions that support learning. 

**Figure 1:**
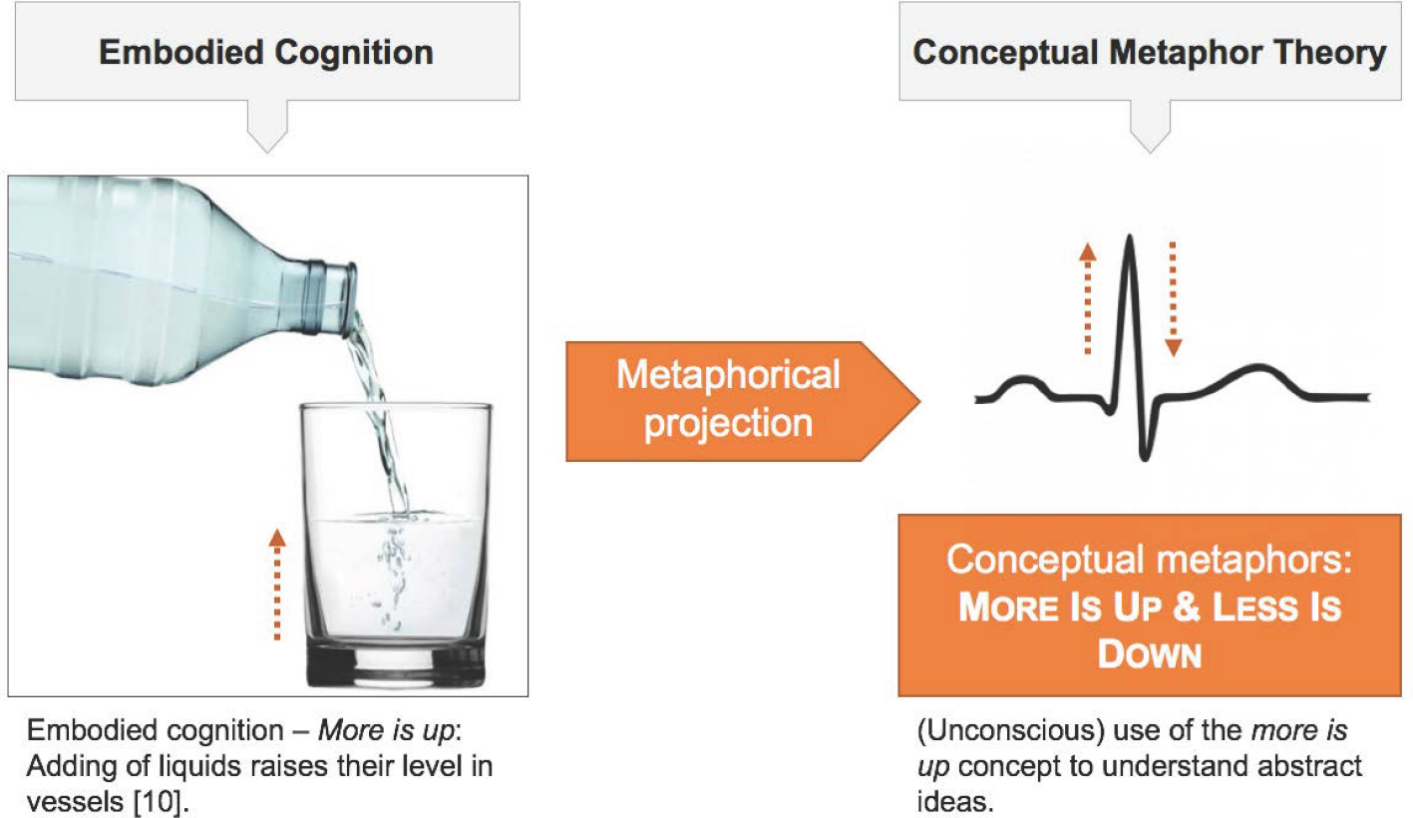
Figure1: Relation of embodied cognition and metaphorical understanding of abstract concepts. The experientially based concept *more is up* is unconsciously activated in other more abstract situations in order to construe mental representations. This study is based on the assumption that students identify the curve progression as increase and decrease of excitation.

According to the theory of embodied cognition, we generate embodied concepts by interacting with our physical and social surroundings. These embodied concepts constitute the core of the cognitive processes available to us. By unconsciously projecting such “cognitive primitives” [11] even abstract concepts can be mentally represented. Embodied concepts which are used to make abstract entities tangible are called conceptual metaphors. This definition of metaphor is reserved to cognitive linguistics and should not be confused with metaphor as defined and used in philosophy and every-day language. Traditionally, the latter two define it as a consciously used figurative expression of poetic language [14]. “Orientational metaphors” [10], amongst others, structure our thinking as can be seen in the example of the *more is up* metaphor [10]. The concept of *more is up* is grounded in embodied experience and can structure the understanding of concepts which are more abstract and harder to grasp. With respect to the concepts of the electrocardiogram it seems reasonable that students are framing the curve progression in terms of this orientational metaphor (see figure 1 (Fig. 1)).

## 3. Research questions

The purpose of this study is to capture the concepts of the physiological ECG that medical students in the study phase of their clinical rotation have and to analyze potentially existing misconceptions. A teaching experiment was developed in order to test an instructional task exploratively. The following research questions are of interest:

1. Which concepts do medical students construe with regard to the physiological ECG and which conceptual metaphors structure their understanding?
2. Which concepts do medical students construe with regard to the physiological ECG while interacting with the theory-based teaching experiment?

## 4. Research design and methods

Conceptions are defined as cognitive constructs reconstructed from people’s utterances. These constructs can be ascribed to individuals according to the context in which the utterances were made. The aim of reconstructing students’ conceptions from their subjective utterances makes a makes a qualitative and explorative research approach necessary. The Model of Educational Reconstruction (MER) [15], which is widely established within science education research, serves as frame of research. Firstly, the learners’ ideas related to the subject matter are analyzed against the background of possible difficulties in understanding (investigation into students’ perspectives). Then, scientific concepts as found in original publications or academic textbooks are examined from the point of view of their intended instructional impact (clarification of science content) in order to finally develop and explore learning opportunities derived from a reciprocal comparison of the two and to then explore them (analysis, design and evaluation of learning environments).

**Figure 2:**
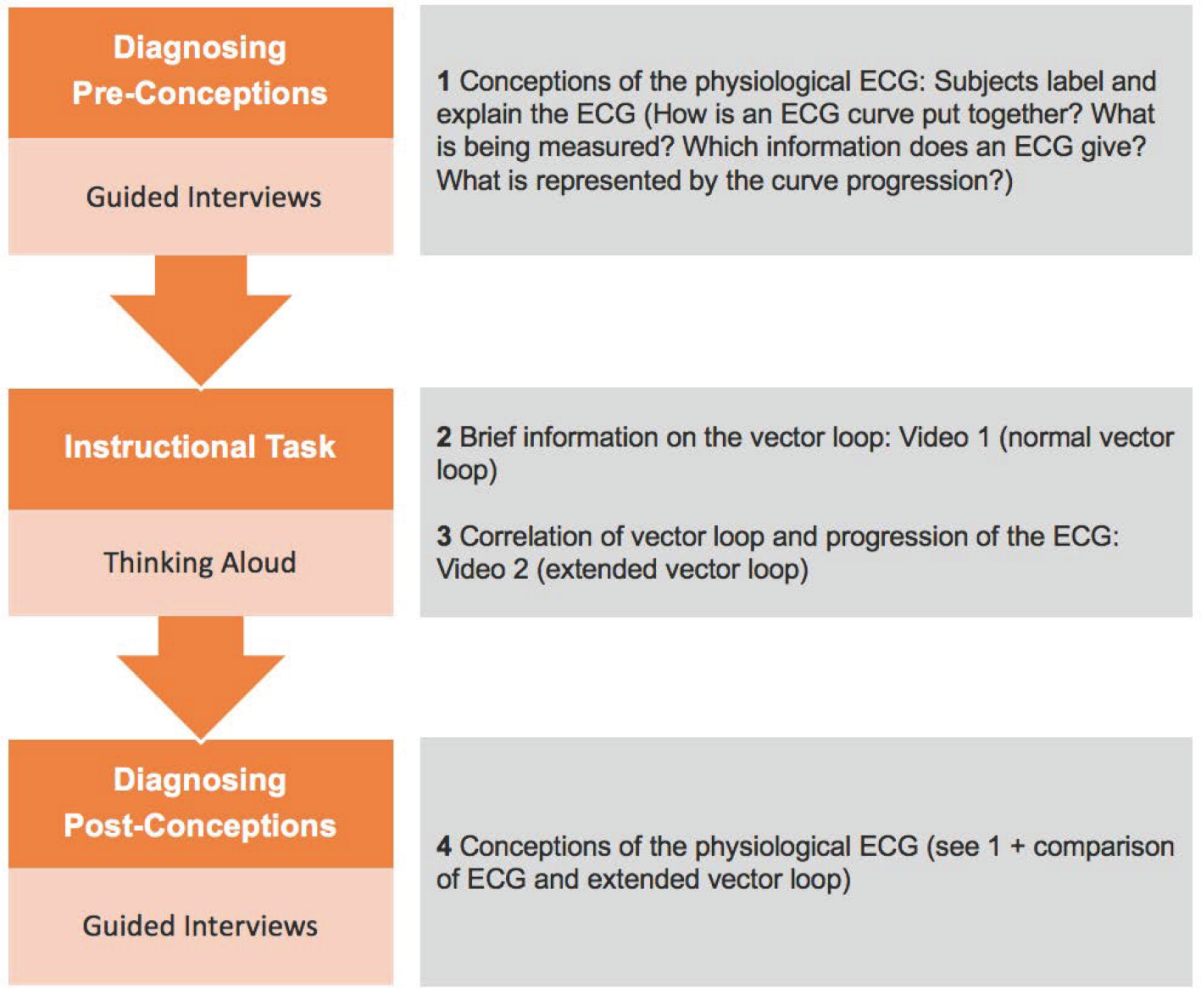
Reciprocal analysis of conceptions und examination of the instructional task – overview of the course of the conducted single-sessions of the teaching experiments.

The methodological setting was oriented towards the teaching experiment [9]. In meetings with individual students (n=10, 5^th^-10^th^ semester), their concepts of the physiological ECG before, during and after the interaction with our intervention (see attachment 1 ) were recorded (see also figure 2 (Fig. 2)). In order to capture possible misconceptions, subjects in the study phase of their clinical rotation were chosen because they have previously taken courses on the physiological ECG. As (mis)conceptions are reconstructed on the basis of conceptual metaphors, an attribution of subjects to specific semesters is not necessary since ideas framed by conceptual metaphors are regarded to be constant within similar cultural environments [11]. 

During the intervention, subjects were asked to think aloud [16] in order to allow close analytic access to the individual thougt processes while being engaged in the animation.

Subjects took part in guided interviews on the ECG before and after the intervention, so that individual pre-conceptions and learning effects could be reconstructed [7]. These meetings lasted 45 to 60 minutes. All videoed statements were evaluated using qualitative content analysis [8] and systematic metaphor analysis [13]. To ensure the intersubjectivity of the research results and their interpretation, the entire data evaluation was based on consensual and argumentative validation in a two-step procedure: The individual transcripts were examined by the author of this study to identify the conceptual metaphors employed. Afterwards, a member of the research team re-examined the transcripts on the basis of the category system compiled in the first step.

After establishing consensus within the research team on the reconstructed metaphors found, the argumentative validation of the data took place in the research group of the Institute for Natural Science Education of Leibniz University Hanover. All subjects were informed about the methodological procedure in detail and gave their consent. The data were anonymized, a matching of citations to the corresponding person is not possible because pseudonyms are used.

## 5. Results

### 5.1 Misconceptions of the physiological ECG

Two subjects showed scientifically correct conceptions during the entire course of the study. Their statements will therefore not be further examined. Eight subjects showed to have scientifically misleading conceptions which will be presented sorted into categories. The exemplary citations, which we chose and will explain in detail, were collected during the phase of the qualitative content analysis. The metaphorical concepts drawn from this are the same as the ones from the “individual structuring” [8] at the end of the content analysis.

#### 5.1.1 Metaphorical concept I: The higher the curve, the greater the excitation [6]

**Exemplary statements (prime examples)**

**Karla**

(27-31): *”*[The excitation in the ECG is greatest]* there, where the peak is the highest.“*

(36-41): *“I would *[mark the lines]* of the depolarisation and repolarisation of the atria. The atria are completely depolarized here, I would say (*marks the maximum of the P-wave*).”*

**Justus**

(147-158): *”This is the P-wave. The other is the P-wave, too – the upward and downward deflection of the P-wave. As this is the excitation of the atria, I interpret this as the increase and decrease in excitation of the atria.”*

**Explanation and metaphor analysis **

Six subjects interpret the rise and fall in the curve progression as an increase and decrease in excitation. The curve maxima are considered as the times of highest excitation. The meaning of the abstract curve of the ECG is mentally represented as the orientational metaphor *more is up* or *less is down* respectively.

This kind of interpretation of a physiological ECG is misleading because the curve maxima (peaks of P-wave and R-wave) are erroneously understood to be the times of complete excitation of the atria or the ventricles.

A further educational challenge is the fact that the basic relation between the ECG curve and the spatial progression of excitation is not recognized.

**Individual structuring (metaphorical concept)**

*More Is Up *I – The higher the curve, the greater the excitation.

##### 5.1.2 Metaphorical concept II: The higher the curve, the more cells are excited [3]

**Exemplary statements (prime examples)**

**Justus**

(109-142): *“I would say that here *[points to the peak of the R-wave]* the excitation is the greatest. There are, as we know, different phases: systole and diastole. Systole is the phase of ejection, diastole the phase of re-filling. And the excitation is probably greatest during the systole, when the highest possible number of muscle cells in the heart (…) are excited and that is the case at the peak shortly before the systole.”*

**Explanation and metaphor analysis**

Justus thinks that the peak of the R-wave correlates with the maximum number of excited heart cells. The *more is up* metaphor structures the understanding here, too. In this case, the curve progression (up/down) is being connected to the number of excited cells (many/ few). From the point of view of educational research this conception is to be regarded as a learning obstacle as it is scientifically misleading. The highest number of depolarized myocardium cells is not reached until the end of the QRS-complex, and not at the peak of the R-wave.

**Individual structuring (metaphorical concept)**

*More Is Up* II – The higher the curve, the more cells are excited.

##### 5.1.3 Metaphorical concept III: The higher the curve, the greater the contraction [2]

**Exemplary statements (prime examples)**

**Lara**

(54-62):* “Because of the addition of the various vectors which represent the electrical excitation, the contraction is, of course, greatest where there is the highest peak. (…) So, up here at the peak of the R-wave the mass-related highest electrical contraction is also occurring.“*

**Explanation und metaphor analysis**

Lara’s conception is also marked by the employing of the *more is up* metaphor. In this case, the highest peak of the curve is connected with the highest excitation and the greatest contraction of the ventricles. The statements also show that excitation and contraction are semantically the same for Lara. She even speaks of “electrical contraction”. From a medical point of view this conception is problematic because an electrocardiogram usually does not give hints on the cardiac contractility. Moreover, the assumed proportionality of curve amplitudes and extent of contraction is scientifically not correct.

**Individual structuring (metaphorical concept)**

*More Is Up* III – The higher the curve, the greater the contraction of the myocardium.

##### 5.1.4 Metaphorical concept IV: Vector sizes represent number of excited cells [1] 

**Exemplary statements (prime examples)**

**Nadja**

(81-99): *“If a few *[cells]* are excited, *[…]* then the vector is relatively small (…).”*

(81-99): *“If more than half *[of the cells]* are excited, (…), then the vector is becoming small again. This is the declining *[part of the P-wave]*.“*

**Explanation und metaphor analysis **

Nadja is mentioning the vectors. However, she thinks that the increase and the decrease of the size of the dipolar vector equals the rise of the ECG curve or respectively its fall. The abstract construct of added dipolar vectors is thus not understood appropriately from the scientific perspective.

Nadja is especially incapable of understanding the relation of the ECG with the vector loop.

She is missing the concept of the vector sum changing extent and direction during the cardiac action. Again, the *more is up* conception is metaphorically used to cognitively grasp abstract information (→ a shrinking vector equals a declining curve). She is another student to whom the aspect of directionality is not conceivable.

**Individual structuring (metaphorical concept)**

*More Is Up* IV – Vector size represents number of excited cells.

#### 5.2 Concepts of the relation of the animated vector loop with the ECG

The theory-based animation focuses on the teaching of the relation of the ECG and the spatial excitation propagation within the myocardium (see attachment 1 ). Two subjects were able to correctly repeat the intention of the learning offers. These were the same two students in who we had entirely scientifically correct conceptions of the physical ECG throughout the course of the study (see 5.1). Their statements will not be examined in the following.

The main focus of the evaluation is the description of the pattern of captured learning effects which could be identified in five subjects. It is documented on the basis of a typical example (subject: Justus). 

In the beginning, the subject explains and understands the ECG curve progression in terms of the orientational metaphors *more is up* and *less is down*. The rise and fall of the graph are interpreted as an increase and decrease of excitation (cf. 5.1.1 and 5.1.2).

Justus realizes in the interaction with the animation that there is a conceptional relation between the graph of the ECG and the vector loop which extends in time.

It is transparent that the animation brings Justus to dissolve the semantical merging of the concepts of spatial and temporal excitation propagation.

*(291-300): “The vector loop and the ECG curve are basically the same. I look at the excitation loop from different angles (…). For one, *[I]* can understand it spatially from different angles and, by protracting it, also temporally.”*

Justus now terms the excitation as something ‘spatial’ and expresses a conception of the ECG curve reflecting the spatial propagation of cardiac excitation within the course of time.

(333-347): “When you look at this, here is the peak of the R-wave and here the peak of the S-wave. You can see that first the depolarization moves in this direction and then in the opposite direction because it goes towards minus. But it is something spatial. The ventricles are then fully excited.”

*(348-355): “In the increase *[of the R-peak]* – in relation to the heart axis, which runs like this – the excitation moves downwards, that is towards my viewpoint, towards me and *[in the downward slope of the P-wave]* the excitation moves away from me.”*

The progression of the ECG is not misinterpreted anymore in terms of the *more is up* metaphor. The understanding is now structured by a new metaphorical concept. The vertical orientation of the curve progression is now understood as the representation of a spatial process (metaphorical concept: *up & down signifies spatial spreading*).

In the course of his interview Justus even realizes his former incorrect view of the R-peak and develops an idea of the electrocardiogram giving information on the spatial excitation progression within the heart. 

*(356-378): “Oh yes, I said that at the high peak the excitation was the greatest. Theoretically, it doesn’t get smaller as such, from my point of view it just changes its spatial orientation. (...) To look at the increase and decrease* [of the ECG]* is not very productive because the amplitude of the peaks is not important primarily, but the viewing angle: Where exactly does the excitation move towards. When I want to look at it in the beginning, it is rather a directional vector, *[it]* gives information on the direction and not on something quantitative like millivolt, while we, of course, measure in millivolt, but for the interpretation of the ECG –educationally speaking – it is primarily important to understand in which direction it moves when I see a rise in this two-dimensional graph.“*

**Figure 3:**
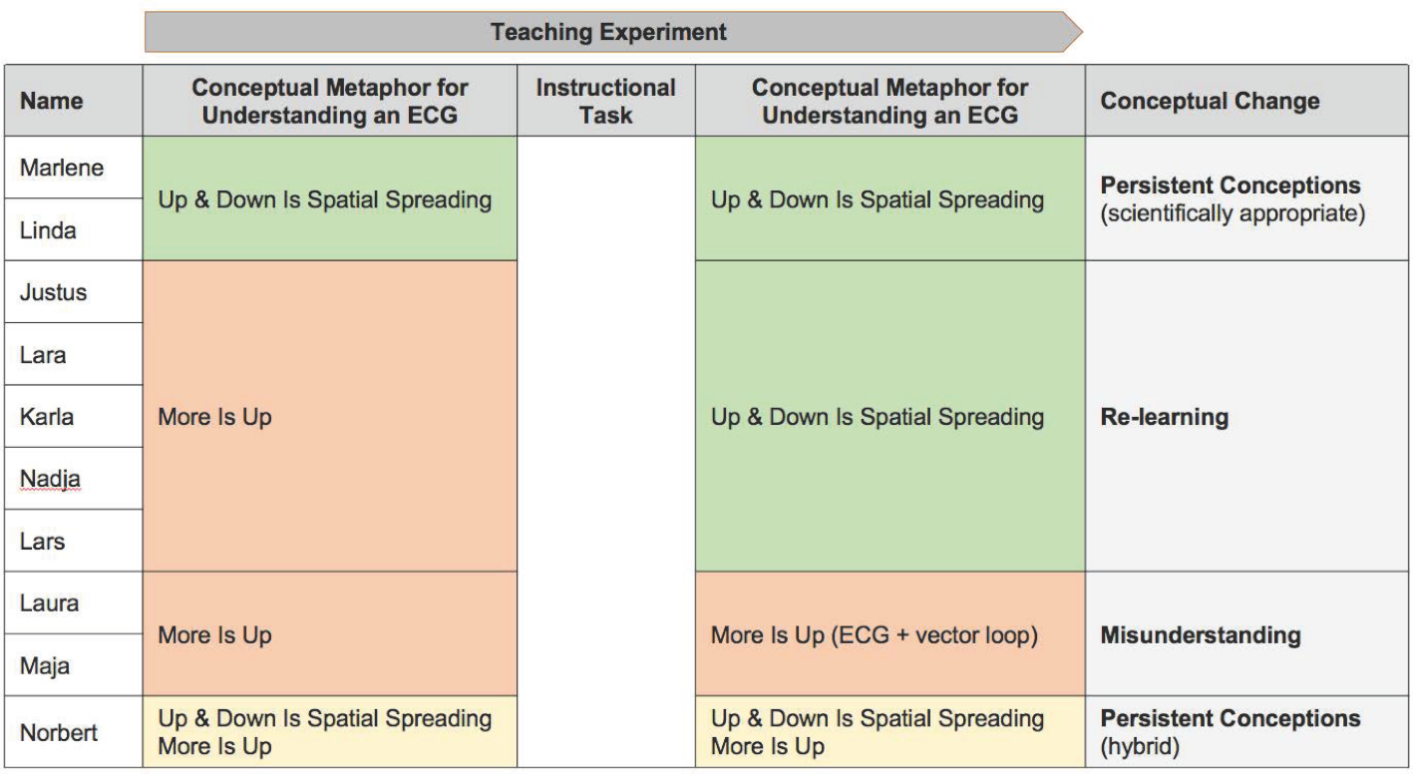
Conceptual metaphors for understanding and misunderstanding the physiological ECG during the teaching experiment

In conclusion, we will take a look at the other three subjects (cf. figure 3 (Fig. 3)). Already in the beginning (in the first phase), Norbert explains correctly that an ECG reflects the spatial propagation of excitation. Rise and fall are interpreted as directions of excitation (metaphorical concept: *up & down signifies spatial spreading*).

*(38-41): “The *[increase in the ECG]* says something about the spatial excitation propagation, that is the electrical activity which moves towards one direction of the electrode [and] away from the other depending on which lead is being projected.”*

However, he also construes misleading conceptions of the ECG curve which can be traced back to the *more is up* and *less is down* concept. 

(26-55): “Then we elevate ourselves to the initial level again and end all this with a slightly bigger elevation than the first one of the P-wave. Then we phase out on the normal line. (…) In principle, the projected excitation is greatest at the peak of the QRS-complex, that is at the R-peak.” 

Even after the intervention Norbert demonstrates hybrid thought constructions. He keeps construing misconceptions which are grounded in the afore described orientational metaphor (metaphorical concept II: the higher the curve, the more cells are excited). Nevertheless, Norbert expresses an idea of the ECG representing directions of excitation.

*(307-321): “The decrease of the P-wave signifies that fewer cells are newly excited in the ventricles than when the graph increases or at its highest peak. This means that fewer cells are excited there than at the highest point of the P-wave. *[At the highest point of the P-wave]* most cells in the ventricle are excited at this time.”*

(391-407): “The key information of the ECG is this: The ECG is showing the electrical excitation propagation within the heart in different directions.”

Norbert admits that he is not able to understand the animated vector loop right away. After a short time of thinking, however, he associates the stretched vector loop with the corresponding ECG parts correctly. 

*(163-190): “Now, it’s understood.* [It was]* drawn statically at first. (…) The first small circle is this part, the second big circle is this part and the small circle then is this part *(pointing first to the P-wave, then to the QRS-complex and then to the T-wave*). *[The inclining part of the R-peak]* should be this large curve.”*

The true intention of the intervention remains hidden to Norbert because he does not realize the relation between the animation and the direction of the excitation propagation represented in the ECG.

During the first part of the teaching experiment it was possible to identify misconceptions Laura had (metaphorical concept: the higher the curve, the more cells are excited). These are also found after the interaction with the animation. We want to emphasize the misconception which came into being by the interaction with the learning offer/animation:

*(590-645): *“[The vector loop] *stretches to the left. This is because the left ventricle is much thicker than the right ventricle and it has much more muscles, so that the loop is bent in this direction and is not – for example – going straight up again.”*

(648-669): “Even though a few go in this direction, the very most go towards this direction and because of that it keeps on going there, that is first in the septum, then it goes to the back, in the ventricles the signal again goes up. And then we’re back to the level of the AV node.”

The subject seems to be imagining that the vector loop is representing the excitation which is moving up and downwards within the heart tissue. The *more is up* metaphor is therefore also used to grasp the abstract vector loop. In this case the animation leads to a misleading conception because the abstract idea of the vector loop cannot be understood in terms that would help to understand the ECG. 

Maja’s statements show that she can label the phases of the ECG correctly. Looking at the data available there are no hints, however, to whether she has got a conception of the spatial excitation propagation represented by the ECG. She also construes scientifically inappropriate conceptions to which verticality again serves as embodied experience. 

*(33-69): “Theoretically, you could say *[that]* here *(points to the peak of the R-wave)* the excitation is the greatest because the peak is the highest.”*

After the interaction with the animation she shows a positive reaction in her comment:

*(136-174): “Oh, I’ve never seen this before. How cool is this?! Yes, of course, *[it]* totally makes sense, when you pull this together, but draw the heart loop like this. It almost looked like a normal ECG. Well, this is news to me. It almost looks identical.”*

Maja indeed is able to match the parts of the stretched vector loop with the corresponding section of the ECG. The statements at hand, however, suggest that she understands the vector loop in terms of the *more is up* metaphor. Thus, this leads to a construction of a scientifically inappropriate idea. 

*(477-498): “Up here *(points to the vector loop)* we would have the beginning of the excitation of the ventricles. This would probably exactly represent the section of the Q-peak. You can see *[the]* ascending* [and]* descending *[section]* here (*points to the corresponding segment in the vector loop*).”*

## 6. Discussion

**Figure 4:**
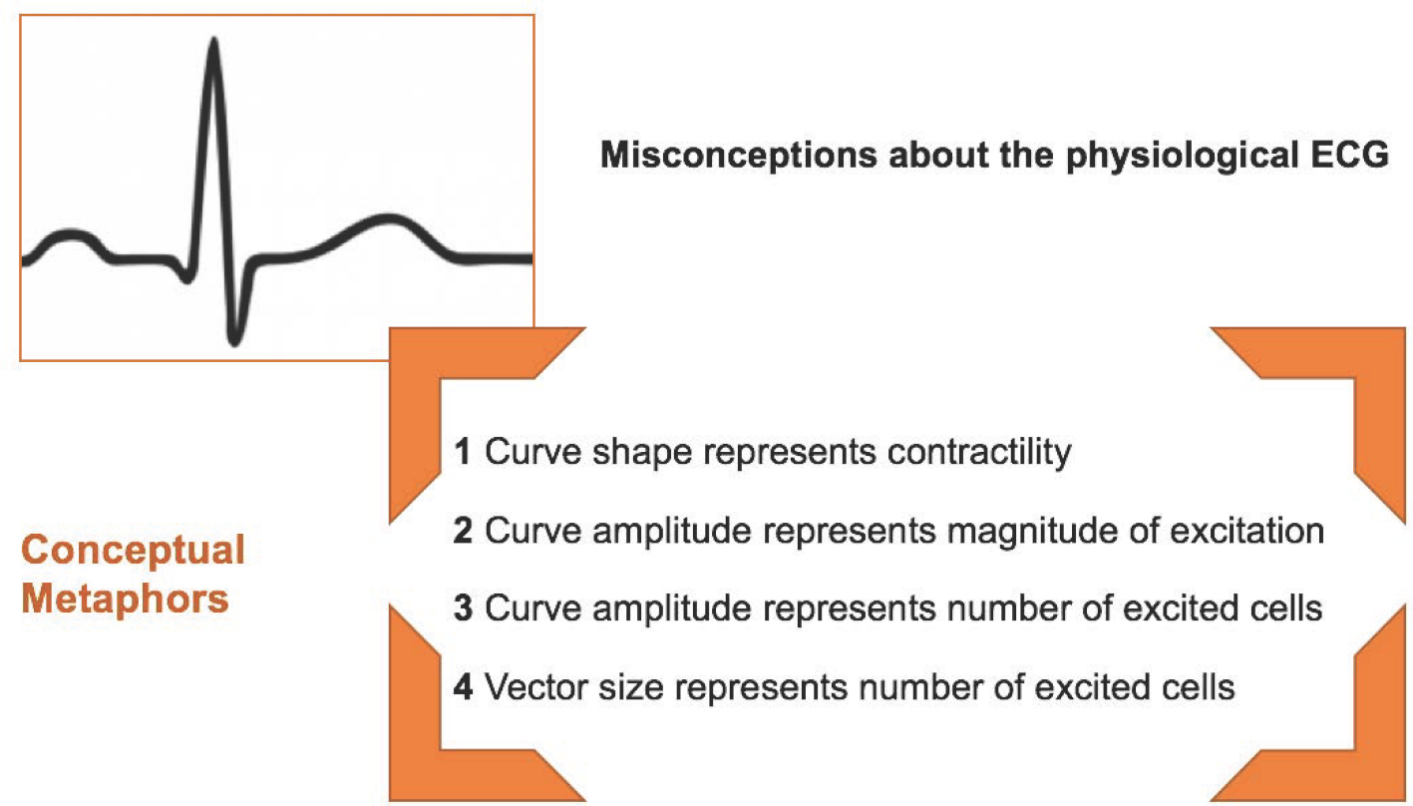
*More Is Up* – metaphorical conceptions impeding an appropriate comprehension of the physiological ECG.

With our teaching experiment, it was possible to identify the misconceptions medical students had construed of the physiological ECG. Using conceptual metaphor theory, the identified misconceptions could be traced back to the misleading *more is up* metaphor. Typically, the curve progression is merely understood as a representation of the increase or decrease of electric activity within the myocardium. The progression of the curve, however, could not be associated with spatial or temporal aspects of cardiac excitation. Hence, the initial hypothesis is supported; or can be rather specified: First, the progression of the curve can be understood as a representation of the cardiac contractility. Second, different amplitudes of the curve can be understood as representations of the magnitude of cardiac excitation or, third, of the number of excited cells. Finally, even the vector size can be understood as a representation of the number of excited cells (see figure 4 (Fig. 4)). 

Because of the limited number of participants of this study, the listing of conceptual metaphors impeding the understanding of ECGs is certainly not intended to be exhaustive. Due to the research design, the sample does not offer representative data. Hence, the results cannot be generalized beyond the test subjects. However, referring to conceptual metaphor theory, misconceptions of a similar type are expected to appear in larger samples, too. Orientational metaphors are assumed to arise from grounded experiences with our physical environment, which are similar to all human beings [11]. 

**Figure 5:**
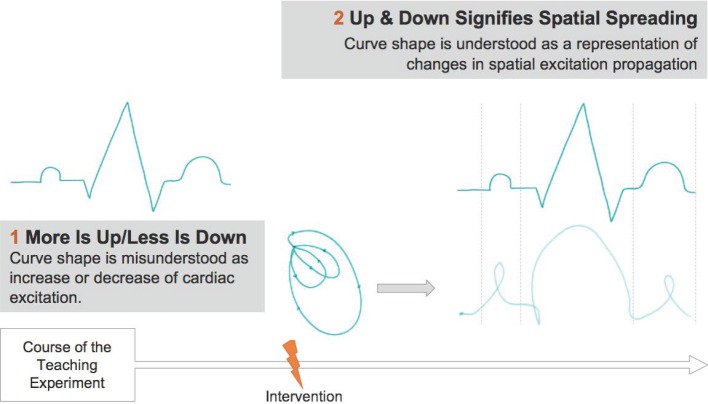
Interacting with the instructional task generates a new conceptual metaphor [2]. As the analysis of the results of the conducted teaching experiments reveal, some of the students are able to explain the ECG as a two-dimensional representation of spatial excitation propagation.

Furthermore, in this study we explored a theory-guided instructional task which focuses on the correlation of the vector loop and the progression of the ECG. Thereby, we are able to show that some students explain an ECG as a two-dimensional representation of spatial excitation propagation appropriately after having interacted with the animated vector loops (see appendix). From then on, the understanding of the ECG no longer relies on the underlying *more is up* metaphor. Instead, the curve´s rise and fall are comprehended as representational models of spatial excitation propagation (see figure 5 (Fig. 5)). Thus, comprehending the ECG is now structured by a new metaphor (*up & down signifies spatial spreading*).

### Limitations

Due to the research approach, all described learning effects are related to the specific situation within the teaching experiment. The learning effects can be explained epistemologically. However, all results have to be seen as descriptive data as they cannot be traced back causally to the instructional task. Predictions on persisting memory performances in regard to the learning effects cannot be done, either. 

The study aims at describing possible conceptual changes which may occur by interacting with the instructional task. But again, it has to be taken into account that any results about the instructional effect cannot be generalized beyond the sample.

The results also demonstrate cases of conceptual misguiding when the *more is up* metaphor is continuously used to comprehend the abstract vector loop. In one case, for instance, the vector loop is understood as the real path of excitation propagation within the actual myocardium. Conceptual metaphor theory can again explain these findings: Our experience of physical objects and substances provides a bold basis for understanding abstract concepts. Thus, we may think about abstract concepts by employing conceptual structures acquired in our experience with the physical and social environment. So-called “ontological metaphors” [10] structure our comprehension of things without clearly distinctive properties. Hence, the abstract vector loop can be imagined as the real path of excitation through the human heart. An abstract process (cardiac excitation) is hereby comprehended as a quasi-material entity moving within a conceivable and real room (the heart). Such kind of conception certainly has to be defined as an obstacle to gaining suitable ideas of cardiologic phenomena. Moreover, we have to assume that such a misconception makes the understanding of pathologic ECGs more difficult. 

Misconceptions are suitable to be used within medical education as contrasting ideas in lectures or tutorials. Again, these findings about possible misconceptions resulting from the teaching experiment are limited to the examined sample. They can neither be generalized, nor make claims of identifying possible other kinds of misconceptions.

## 7. Conclusions

This study aims at analyzing medical students’ conceptions of the ECG. Misconceptions could be identified and traced back to four metaphorical concepts. Considering these misconceptions can contribute to taking action in order to align preclinical cardiologic education. For instance, these misconceptions are suitable to initiate cognitive conflicts by comparing them with appropriate concepts [17] within lectures or tutorials. 

The theory-guided instructional task, which focuses on the relationship between the vector loop and the ECG, constitutes an approach to directly improve medical students’ comprehension of the spatial and temporal aspects of cardiac excitation represented in the ECG. As a consequence, the animation of the extended vector loop can be used as a helpful task to revise misconceptions. It can also be used in lectures during the preclinical education in order to prevent the arising of such misconceptions. The approach can be easily integrated within lectures and tutorials (students also could draw the sketches themselves). 

Cases of conceptual misunderstandig, however, are also shown as results of the conducted teaching experiments. Since students can understand both, ECG and vector loop, by using the *more is up* metaphor, this possible misconception could also be utilized as a contrasting approach [17] within the learning process. 

Using a qualitative-explorative research design and especially the conceptual metaphor theory have been beneficial in analyzing conceptions as well as misconceptions of the ECG. Thus, insights into hitherto unexplored aspects of teaching and learning cardiologic basics have been obtained. 

Moreover, the results lead to further research questions. It will be of interest, for example, if the described learning effects are lasting and, if so, how they are made to be lasting. Furthermore, the question is, whether they contribute to the understanding of different kinds of pathological ECGs. 

With regard to analyzing potential obstacles in the diagnosis of cardiac diseases, our research approach could also be used to examine (mis)conceptions of pathological ECGs. It could also be of interest, if modulated versions of the extended vector loop acted as instructional tasks to help students interpret pathological ECGs (e.g. bundle branch heart blocks with deviations in the QRS-complex). 

Finally, these findings can form a data basis for further quantitative research (e.g. comparative studies) on the efficacy of the instructional task. Being aware of the identified conceptions and misconceptions could support researchers to develop tests of knowledge purposefully and, in consequence, to retest the qualitative findings in representative samples.

## Acknowledgements

Special thanks go to Theresa Sethmann and Anja Schirmer for supporting part of this research work and also to Prof. Dr. Harald Gropengießer for scholarly exchange. Moreover, I thank Prof. Dr. Tobias Raupach and the study group for medical education research at Georg-August-University (Göttingen) for their feedback. Finally, I give thanks to the reviewers for their constructive suggestions on the manuscript. 

## Audiovisual material

Audiovisual material for this article is available from the Dryad Digital Repository: [https://doi.org/10.5061/dryad.f19p512] [18]

## Competing interests

The author declares that he has no competing interests. 

## Supplementary Material

Attachment 1
