# Supplementary material for: A qualitative study on the development and rectification of advanced medical students’ misconceptions about the physiological electrocardiogram (ECG)
Source: GMS J Med Educ. 2019 Nov 15;36(6):Doc72. doi: 10.3205/zma001280 (PMC6905362; doi:10.3205/zma001280)
Supplement: Attachment 1 [file JME-36-6-72-s-001.pdf]

Developing the Instructional Task

The ECG represents spatial propagation of cardiac excitation two-dimensionally. Therefore, the progression of the curve amplitude correlates to the specific excitation propagation within the three-dimensional space.

Conceptual Blending – Mapping Spatial and Temporal Processes

Understanding this hidden fact requires people to envision two processes simultaneously. Combining the three-dimensional propagation of cardiac excitation with the course of time constitutes a sophisticated cognitive task. Assisted by two simulations this kind of mapping can be illustrated:

1. Preparation (Repetition of Content Knowledge): Representing the Propagation of Cardiac Excitation Two-Dimensionally (Vector Loop)

The following clip shows the loop-shaped graph which occurs in the course of the excitation. It can be depicted as the representation of the chronological change of the vector sum, which is projected onto one specific plane (frontal, sagittal or horizontal). Thereby, students are reminded of the basic information of the vector loop.

Video (Clip 1): https://doi.org/10.5061/dryad.f19p512

2. Instructional Task (Intervention): Representing the Propagation of Cardiac Excitation in the Course of Time

The abstract phenomenon of time is understood by conceptual metaphors [18]. For example, time can be comprehended as a substance-like commodity (*Today, there is only little time left.*). We can also understand time as a movable entity (*Time has passed very quickly again*). The latter metaphor is used within the instructional task: In the following clip, time will run through the described process of cardiac excitation propagation. Hence, this animation contains the theory-guided approach to evoke re-learning processes.

Video (Clip 2): https://doi.org/10.5061/dryad.f19p512

The extended vector loop, which is the result of the second clip, is similar to the ECG. Students are prompted to be able to capture the idea of spatial cardiac excitation propagation, which is otherwise hidden in the ECG.

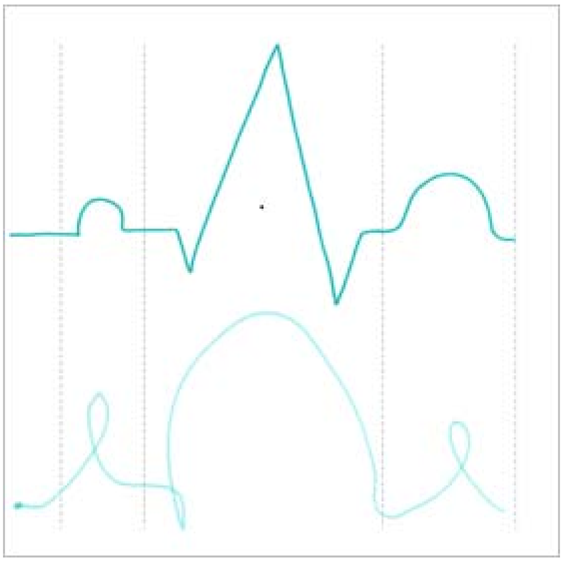

Figure 6: Similarities of the ECG and the extended vector loop visualize the relation between the ECG curve and the currently varying vector sum.
